# Bipolar spectrum disorders in neurologic disorders

**DOI:** 10.3389/fpsyt.2022.1046471

**Published:** 2022-12-21

**Authors:** Anna Digiovanni, Paola Ajdinaj, Mirella Russo, Stefano L. Sensi, Marco Onofrj, Astrid Thomas

**Affiliations:** ^1^Department of Neuroscience, Imaging and Clinical Sciences, “G. D’Annunzio” University of Chieti–Pescara, Chieti, Italy; ^2^Center for Advanced Studies and Technology (CAST), “G. D’Annunzio” University of Chieti–Pescara, Chieti, Italy

**Keywords:** bipolar spectrum disorders, neurology, psychosis, dementia, immunity

## Abstract

Psychiatric symptoms frequently predate or complicate neurological disorders, such as neurodegenerative diseases. Symptoms of bipolar spectrum disorders (BSD), like mood, behavioral, and psychotic alterations, are known to occur – individually or as a syndromic cluster – in Parkinson’s disease and in the behavioral variant of frontotemporal dementia (FTD). Nonetheless, due to shared pathophysiological mechanisms, or genetic predisposition, several other neurological disorders show significant, yet neglected, clinical and biological overlaps with BSD like neuroinflammation, ion channel dysfunctions, neurotransmission imbalance, or neurodegeneration. BSD pathophysiology is still largely unclear, but large-scale network dysfunctions are known to participate in the onset of mood disorders and psychotic symptoms. Thus, functional alterations can unleash BSD symptoms years before the evidence of an organic disease of the central nervous system. The aim of our narrative review was to illustrate the numerous intersections between BSD and neurological disorders from a clinical-biological point of view and the underlying predisposing factors, to guide future diagnostic and therapeutical research in the field.

## Introduction

Bipolar spectrum disorders (BSD) are psychiatric conditions characterized by unstable mood and the presence of alternating manic/hypomanic and depressive episodes. BSD markedly impact the global quality of life of patients (1). Interestingly, the comorbidity of BSD with neurological diseases has been extensively described (2). Shared genetic predisposition and neurodevelopmental alterations are a common ground for developing different neuropsychiatric conditions.

According to the fifth edition of the Diagnostic and Statistical Manual of Mental Disorders (DSM-5) BSD are classified into three primary patterns: bipolar disorder (BD) I, II, and Cyclothymia (3). BD I is the classical “manic-depressive disorder” characterized by the occurrence of at least one manic episode, i.e., “a period of abnormally and persistently elevated, expansive, or irritable mood and abnormally and persistently increased goal-directed activity or energy” (3, 4). During the manic phase, BD patients frequently suffer from impulse control disorders (ICDs), like gambling, hypersexuality, shopping sprees, bulimia, aggressive driving, and hoarding (5). Most subjects with BD I also experience major depressive episodes in their lifetime, although its presence is not a required diagnostic criterium. BD II diagnosis requires at least one episode of major depression and at least one hypomanic episode that, differently from manic episodes, are not severe enough to cause severe impairment in social or occupational functioning or necessitate hospitalization (3). The Cyclothymic Disorder is diagnosed when, for at least 2 years, the subject has experienced hypomanic and depressive periods without fulfilling the criteria for a full-blown episode of mania, hypomania, or major depression (3).

Bipolar disorder categorization has mainly been conceptualized by American authors. However, French and German psychiatrists have the merit to have drawn attention to Cyclic Psychoses largely before the American studies (6–12). Also, European studies attempted to extend the DSM categorization from three to six clinical syndromic entities (13, 14). These six conditions range from hypomania, not interfering with adaptation to social life and career goals, to patterns associated with neurodegeneration. BD type VI, in particular, is characterized by cognitive decline and mood instability and may be interpreted as a late-onset BSD. In these patients, an accurate evaluation of premorbid personality and familiar history of BSD may help define the therapeutic strategy since mood stabilization may be more effective than antidepressants and acetylcholinesterase inhibitors (14).

In a MEDLINE (Medical Literature Analysis and Retrieval System Online) search, “Bipolar Disorders” AND “Dementia” keywords generate more than 1,400 results, “Bipolar Disorders” and “Parkinson” more than 700 papers, “Multiple Sclerosis” and “Bipolar Disorders” above 200 papers, and finally the combined search with Epilepsy finds about 1,000 papers.

**FIGURE 1:**
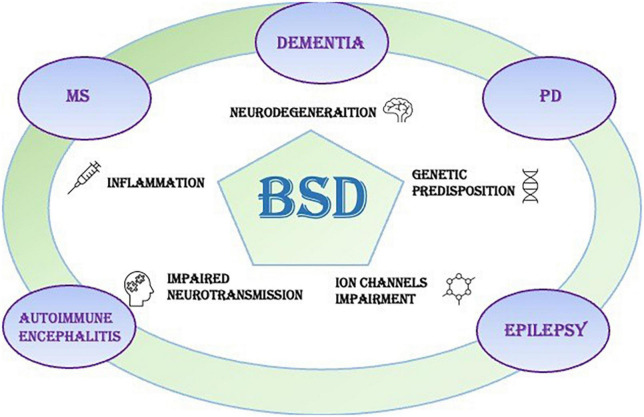
Bipolar spectrum disorders (BSDs) frequently occur in neurological disorders of the central nervous system, like dementias, Parkinson’s disease (PD), multiple sclerosis (MS), autoimmune encephalitis (AE), and epilepsy. The pathophysiological triggers of the neurological symptoms can play a role also in the onset of mood disorders like BSD.

The present review analyzes the literature on the co-occurrence of BSDs and Neurological Disorders to understand whether different comorbidity patterns indicate clusters, endophenotypes, or statistical overlaps (Figure 1). We omit from the review the rare genetic and metabolic disease, where coexistence of psychiatric and neurologic symptoms, together with signs of medical and laboratory dysfunctions, are core elements for the diagnosis, like Wilson’s disease (15).

## Bipolar spectrum disorders in neurologic disorders

### BSD and dementia

Compared to other adult psychiatric disorders BSD, are associated with an increased risk of developing neurodegenerative disorders and cerebrovascular diseases (16). Several studies concur in showing that dementia risk in BSD patients is remarkably higher (20–25%) than in the general population (7%) (17). In some cases, BSD present a progressive course, with an increasing number of hospitalizations, poor response to treatment, and steady cognitive decline in the late phases of the disease (16). However, some other studies suggest that early alterations of cognitive functions are part of the BSD phenotype (17–19). A remarkable inter-subject heterogeneity is observed in terms of compromised cognitive domains (20), converging toward a more prominent impairment of verbal memory, attention, executive functions, and visual memory (20). Some data suggest that cognitive decline correlates with the clinical course of BSD, for instance, with the number of altered mood episodes – in particular the maniac ones –, the occurrence of hospitalizations, and the duration of illness (16, 21, 22). It has been hypothesized that mood swings trigger cumulative damage in neural cells and then feed a vicious circle causing more manic or depressive episodes (23). Inflammation, genetic predisposition, and neural vulnerability associated with the disease duration, and the number of mood episodes may favor accelerated brain aging in BSD (1). This mechanism may explain the coexistence of the two conditions, neuropathological findings in the brain of BSD patients and imaging signs of cortical atrophy associated with cognitive decline. Moreover, some dementia subtypes are characterized by early mood and behavioral alterations (16), as later detailed.

#### Frontotemporal dementia

##### Clinical overlap

The frontotemporal dementia (FTD) spectrum encompasses several neurodegenerative entities that share prominent damage of the frontal and temporal lobes (16). According to the clinical presentation, FTD can be categorized into three subtypes: the behavioral variant (bvFTD) and three forms of Primary Progressive Aphasia (APP) (logopenic, semantic, and non-fluent/agrammatic) (24). Behavioral features appear consistently also in patients whose phenotype is showing prominent APP features, especially in the semantic variant (25). From a genetic standpoint, FTD is known to be highly inheritable and related with several genes, such as TAR DNA-binding protein (TDP), progranulin (GRN), chromosome 9 open reading frame 72 (C9orf72), *fused in sarcoma* (FUS) and some other rare disease genes (26). Patients with bvFTD may be diagnosed as suffering from psychiatric conditions (Major Depressive Disorder – MDD, BSD, Schizophrenia – SCZ) more often than dementia patients affected by Alzheimer’s disease (AD) or other FTD subtypes (16, 27). Due to symptoms like disinhibition or increased talkativeness and distractibility, early-onset FTD cases must be differentiated from late-onset BSD (28). From a neuropsychological standpoint, bvFTD is characterized by deficits in executive functions, verbal memory, and emotional processing (28). All these alterations may also be found in subsets of BSD patients, especially late in life (28). Psychosis is the most common psychiatric symptom in bvFTD patients, occurring in almost the 50% of cases (16), mainly as delusions and hallucinations. Visual hallucinations, which were initially considered a feature of dementia with Lewy bodies (DLB), are described in C9orf72 and GRN genetic variant carriers (25). Manic or hypomanic episodes, in the absence of cognitive alterations, may appear years before other symptoms of bvFTD and be a prodrome of dementia, possibly indicating an anterior temporal involvement in the disease (16). On the other hand, patients with late-onset bipolar disorder may exhibit a non-progressive “FTD Phenocopy Syndrome” (28), defined as a syndrome that initially meets the criteria for possible FTD but remains stable after 3 years and does not show neuroimaging abnormalities. Furthermore, a distinct subtype of post-BD dementia has been described, with clinical features similar to bvFTD (29).

##### Biomarkers

Typical imaging findings of full-blown FTD (cortical atrophy of the frontal and temporal lobes) may not be present in the disease’s early stages. Also, certain degrees of cortical atrophy can be found in the late course of BSD (28, 30, 31), even though less prominent than in FTD, thereby representing a further complication for the differential diagnosis. 18-Fluorodeoxyglucose-positron emission tomography (FDG-PET) is employed to confirm the diagnosis of bvFTD over BSD, since brain hypometabolism is an early marker of neurodegeneration. Still, several false positive and false negative cases have been described. Also, unspecific hypometabolism, especially in the dorsomedial prefrontal cortex and the superior temporal gyrus, can be found in BSD patients (28). Only follow-up visits can discriminate between the two conditions in these cases.

##### Converging biological pathways

Both disorders present substantial degrees of inheritance, estimated to be 25% for FTD and 70% for BSD (28). Some genetic loci seem relevant in FTD and BSD (e.g., C9ORF72 and progranulin) (16, 28). For instance, a case of late-onset BSD presenting the C9ORF72 hexanucleotide expansion (32) has been described as well as cases presenting progranulin mutations (33). Moreover, the variability of the GNR gene seems to be related to the susceptibility to develop the BD-I subtype of BSD (34). Phenotypical similarities between late-onset BSD and early bvFTD, as well as the results of GWAS studies, prompted the hypothesis of common molecular mechanisms that cause a neuropsychiatric continuum between the two conditions. Inflammation, a well-known predisposing factor for BSD, has been studied when exploring the association between BSD and FTD. For instance, increased levels of interleukin-6 (IL-6), tumor necrosis factor-alpha (TNF-α), and progranulin have been recognized as common inflammatory markers in BSD and FTD (16). Moreover, some human leukocytes antigen (HLA) haplotypes have been related to both BSD and FTD (16).

All these data, taken together, prompted the definition of a new framework in which psychiatric and neurologic conditions – so far considered compartmentalized nosological entities – are described as a continuum epiphenomenon of staged accumulations of structural and functional damage of the cerebral tissue (accelerated brain aging).

#### Alzheimer’s disease

##### Clinical overlap

Although AD, in its early stages, may present with mood disorders, mainly depressive episodes, other clinical features of BSD are not typically present in AD. However, both conditions are characterized by increased agitation and aggression, affective lability, dysphoria, apathy, impaired self-regulation, and, in some cases, psychosis (35, 36). Of note, the frontal variant of AD (37) is featured by euphoria, disinhibition, and other FTD-like symptoms. Genetic, early-onset AD (EOAD) – e.g., related to PSEN1 mutation – can present with frontal dysfunction (38) and, since it affects subjects in their 30s–40s, may require a differential diagnosis from psychiatric disorders including BSD. Finally, some cases of BSD ultimately lead to cognitive and independence decline, as suggested by Akiskal’s classification (type VI) (13).

##### Biomarkers

BSD and AD share common structural and functional alterations in the hippocampal area. In AD, the hippocampus shows the most prominent and early features of neurodegeneration (37, 39). Alterations in the functional activity of the hippocampus have also been associated with the presence of emotional and cognitive impairment in several mental disorders, including BSD (40). As expected, age-related structural changes in the hippocampus are found in BSD patients (41). Interestingly, the hippocampal volume is also reduced – compared to controls – in young BSD patients naïve to treatment. At the same time, it is relatively increased in lithium-treated BSD patients, thereby suggesting a potential neuroprotective role for the compound (42). Thus, long-term untreated BSD patients may exhibit structural neuroradiological findings that mimic hallmark AD-related neurodegeneration.

##### Converging neuropathological pathways

From a molecular point of view, BSD and AD share some epigenetic features. These include the hyper/hypo methylation of some promoter regions of the DNA that drives to impaired expression of crucial proteins (e.g., neurotrophic factors and metabolizing enzymes) (41). Accordingly, the expression of the Brain-Derived Neurotropic Factor (BDNF) is decreased in old BSD patients (43), as well as in AD (44). Moreover, BSD patients show increased levels of AD-related amyloid-β (Aβ) (45, 46), with a positive correlation with the number of affective episodes (46). In contrast, no significant changes in cerebrospinal fluid (CSF) levels of tau protein have been found (47). No AD-related CSF alterations have been observed in BSD patients, but some impairment of the Aβ clearance during affective episodes was found, suggesting an association between symptoms and brain amyloidosis in BSD (48). Moreover, dementia in BSD is not associated with the e4 allele of apolipoprotein E (APOE*4), a well-established risk factor for AD (48).

#### Parkinson’s disease and dementia with Lewy bodies

##### Clinical overlap

Bipolar spectrum disorders, Parkinson’s disease (PD), and DLB share several clinical features, such as the presence of ICD, and psychotic symptoms (49–52). For instance, hallucinations and delusions are typical of DLB and PD (49, 50) but also of the manic phases of BSD (51). While visual modality is the most common presentation of hallucinations in all three conditions (49–51), delusional content may differ, with paranoid delusions more commonly found in DLB and grandiose types in BSD (53). Impulsivity is a core feature of BSD that leads to engagement in risky behaviors, especially during the manic/hypomanic phases (3, 54) and is also present in parkinsonian patients (55). However, for decades, parkinsonian ICDs have been attributed to iatrogenic side effects of Dopamine Agonist therapies (56–64), before being recognized as a core symptom of the disease, appearing often before any exposition to drug treatments. This phenotype is described in carriers of PRKN and GBA mutations (65–67) leading to PD. Because of the assumed effect of drug treatments, the co-occurrence of BSD and PD has been largely neglected, until large meta-analysis and cohort studies have shown an unquestionable correlation (68–70). Bipolar patients carry a 3.4- to 6.78-fold higher risk of developing PD (68, 69). The risk seems related to the magnitude and frequency of mood shifts (68). The overlap between the two conditions shapes the disease course. Compared to “pure” PD patients, BSD-PD patients, for instance, have been reported to carry higher degrees of neuropsychiatric symptoms like ICD, delusions, or somatizations (5). Furthermore, cognitive impairment may occur earlier in these BSD-PD patients, who also typically exhibit worst outcomes after a Deep Brain Stimulation surgical procedure (71).

##### Biomarkers

The link between BSD and PD has been challenged, by some authors, by calling into question the potential occurrence of iatrogenic parkinsonism in lithium (72) – or valproate (73) – treated patients. However, the high prevalence of dopaminergic transmission impairment at molecular neuroimaging (20%), in bipolar subjects (72), confirm underlying neurodegeneration in many cases. Furthermore, several fMRI studies have documented, in BSD patients, a lack of deactivation during tasks of cortical regions belonging to the default mode network (DMN) [namely the medial prefrontal cortex (mPFC) (74–76), and posterior cingulate cortex (PCC) (77)] along with a decreased activation of dorsolateral prefrontal cortex (DLPFC) (78). This condition, considered a robust indicator of BSD, may represent the hallmark of a deranged connectivity [possibly due to white matter damage (79)] between the DMN and the attentional networks (a condition also known as “corticolimbic dysfunction”) (78, 80, 81). As a consequence, the reality-checking failure in these patients is impaired. The same findings were described in patients with PD or DLB, consisting of disinhibition of PCC and decreased activation of DLPFC (82, 83). In DLB the activity of PCC is proposed as a diagnostic index, vs. other types of dementia, and is denominated Cingulate Island Sign (82, 83). Therefore, psychosis, in the two disorders, shares a common pathophysiological ground as evidenced by networks dysfunctions (84, 85).

##### Converging neuropathological pathways

The dysregulation of the dopaminergic circuitry – the leading cause of several parkinsonian symptoms – has also been recognized in BSD. In particular, the combined mood/motor fluctuations of PD (e.g., motor and psychic off states vs. dyskinesia and ICD) have been compared to BSD-related mood swings (69, 86). However, while mechanisms by which dopaminergic dysregulations and related neurodegeneration occur in PD are quite clear the BSD pathophysiological cascade is less understood. For instance, the neuropathology of BSD does not account for a unique, distinctive pattern. However, sporadic observations have confirmed the possible presence of Lewy body pathology in bipolar patients (87, 88). Moreover, a decreased parietal concentration of monomeric α-synuclein, possibly reflecting a synaptic impairment, has been observed in bipolar patients who had developed parkinsonism (89). Still, further studies are needed to assess the biochemical and pathological relationships between synucleinopathies and BSD.

### BSD and multiple sclerosis

#### Clinical overlap

Multiple sclerosis (MS) is a chronic autoimmune inflammatory demyelinating disorder of the central nervous system (CNS) with a variable and unpredictable course (90, 91). Mood disorders are commonly the most reported neuropsychiatric comorbidity in MS. These are also associated with lower treatment compliance, poor outcomes and functional status, and overall decreased quality of life (90, 92–94). An increased risk for psychiatric disorders is recognized even before a definite diagnosis of MS is made (OR = 1.4, IC 1.05–1.88) (95). The lifetime prevalence of BSD is higher in people with MS than in sex- and age-matched controls, and estimated to range from 0 to 16.2% in different population-based studies (91, 96–98). BSD also affect the pediatric MS population with a prevalence of 3.57% (99). In a recent meta-analysis on MS, a higher BSD prevalence was found in the Americas than in Europe (90, 98, 100).

#### Biomarkers

White matter abnormalities (WMa) are a typical MS neuroradiological finding. In BSD patients, WMa are also common. They occur, predominantly in the right posterior temporoparietal and left cingulate regions, along with thinning of the cortical gray matter in the frontal, temporal, and parietal regions of both hemispheres (101, 102). MS lesions in brain areas, critical for the control of affective functions, could be a substantial contributing factor toward an increased diagnosis of BSD in this population (98, 103, 104).

#### Converging biological pathways

The link between MS and BSD has not been fully explored. However, a possible genetic association involving the HLA region has been suggested in patients with MS and a family history of BSD (98, 105–109). Neuroinflammation is a shared trait between MS and – to a lesser extent – BSD (90). Neuroinflammation is involved in the pathogenesis of mood disorders, as indicated by the *post-mortem* detection of increased protein and mRNA levels of interleukin 1β (IL-1β), interleukin 1 receptor, myeloid differentiation factor 88 (MyD88), nuclear factor-kappa B (NFκB) subunits and astroglial and microglial markers (glial fibrillary acidic protein, GFAP; inducible nitric oxide synthase, iNOS; c-fos, and CD11b) in the frontal cortex of BSD patients (110, 111). In addition, mood symptoms are exacerbated by MS treatments like corticosteroids or baclofen (112). High-dose corticosteroids, in addition to anti-inflammatory effects, can modulate metabolism, cognitive performance, and emotional responses due to the interaction with several neurotransmitters and neuropeptide systems, as well as increasing adrenal responsiveness (113). Thus, while steroids’ capacity to trigger manic episodes is well understood, the mechanism through which baclofen – a myorelaxant – induces manic symptoms is not well identified but may partially rely on its effects on the dopaminergic or serotoninergic systems (114).

### BSD and autoimmune encephalitis

#### Clinical overlap

Encephalitis is an inflammatory condition of the brain frequently caused by either viral or immune-mediated processes. The latter, namely called “autoimmune encephalitis” is due to the production of antibodies against intracellular neuronal proteins (onconeural proteins), or neuronal cell surface/synaptic proteins. The most common forms of AE are those caused by antibodies anti N-methyl-D-aspartate (NMDA) receptor, anti-leucine-rich glioma inactivated 1 (LGI1), anti-contactin-associated protein-like 2 (CASPR2), anti alpha-amino-3-hydroxy-5-methyl-4-isoxazolepropionic acid (AMPA) receptor, anti gamma-aminobutyric acid A or B (GABA-A or GABA-B) receptor, anti IgLON family member 5 (IgLON5), anti dipeptidyl-peptidase-like protein-6 (DPPX), anti glycine receptor (GlyR), anti neurexin-3 alpha and anti myelin-oligodendrocyte glycoprotein (MOG) (115). Encephalitis – limbic variant above all – is characterized by neurologic dysfunction and psychiatric symptoms like irritability, mood disturbance, hallucinations, and personality disturbances, mostly present in anti-NMDAR, anti-GABA-A-R and anti-AMPAR encephalitis (115–118). BSD symptoms can be the only clinical manifestation of AE at the onset and can be attributed only to a psychiatric disorder, thereby leading to misdiagnosis (117, 118). Moreover, antibodies typical of different AE can be found in patients with BSD or other psychiatric disorders without neurological involvement (117, 118). Patients with bipolar disorder frequently present with antibodies against AMPAR, ATPA (H/K Adenosine Triphosphate), CASPR2, GAD65 (Glutamic Acid Decarboxylase), NMDAR and VGKC (Voltage Gated Potassium Channel) complex in serum (116, 119). Almost 67% of patients with anti-NMDAR encephalitis (ANMDARE) experience psychiatric symptoms and require psychiatric care (120–123). After a first “flu-like” phase of the disease, with subtle symptoms such as anxiety, agitation, and short-term memory loss, the following phase is characterized by 1–3 weeks of more severe and debilitating mood and behavioral disturbances, cognitive impairment, and psychosis. Eventually, the full-blown phase consists of dyskinesia, dystonic posture, seizures, autonomic instability, and decreased level of consciousness (121). A subset of patients affected by ANMDARE presents pure psychiatric symptoms during specific periods of their illness, with no neurological symptoms (121). BSD should also be considered in the differential diagnosis of catatonia, which is present in many other medical and neurologic conditions that require prompt recognition and intervention. Catatonia could be the next step of the delirious mania, characterized by an acute onset of manic signs and symptoms, accompanied by a waxing and waning pattern of consciousness (124) and is also a feature of AE dependent on different autoantibodies than the anti-NMDAR, e.g., anti-GABA (115). The case of a patient with a late-onset manic syndrome, followed by a worsening of the level of consciousness, and catatonic features, has been described (124). While, in the initial phase, the symptoms were attributed to BSD, further investigations were consistent with a diagnosis of Non-Convulsive Status Epilepticus and the patient was treated with benefits with immunotherapy, suggesting the probable diagnosis of AE (124). Moreover, also anti-AMPAR encephalitis can be characterized by psychiatric symptoms (125). In a case series of 22 patients with AMPA receptor antibodies psychosis with bipolar features has been described in one patient (125). In literature it has been also reported the case of a 20-year-old woman with Turner Syndrome who presented bipolar mood disorder, psychotic and other psychiatric symptoms, and anti-AMPA receptor encephalitis (126).

#### Biomarkers

In a cohort of 571 patients with ANMDARE, it has been found that the levels of auto-Abs decreased across the manic episode, suggesting a possible role of anti-NMDAR auto-Abs as a biomarker of acute episode (120, 121). Moreover, anti-NMDA receptor Abs have also been detected in serum from ∼10% of healthy controls as well as in patients with pure psychotic symptoms suggesting an autoimmune process as a pathogenetic mechanism in a subgroup of bipolar patients who present increased levels of anti-NMDAR auto-Abs.

#### Converging biological pathways

The imbalance of glutamatergic system is involved in the pathophysiology of neuropsychiatric symptoms, including BSD. Evidence derived from genetic, post-mortem, biochemical, and imaging studies suggest that BSD is characterized by altered levels of glutamate, abnormalities in the NMDAR gene expression, concentration, and functioning (111, 127). ANMDARE is triggered by IgG auto-Abs against the NR1 subunit of the NMDAR. It has been supposed that the early phase of the ANMDARE is related to initial intrathecal penetration and antibody diffusion into cortical and hippocampal gray matter with resulting disinhibition and psychiatric symptoms. In the late phase, a secondary immunologic expansion within the intrathecal compartment could determine the further imbalance of the glutamatergic neurotransmission with an increase of neurologic symptoms (121). The involvement of anti-AMPA receptor antibodies confirm the implication of the glutamatergic system in the pathogenesis of both BSD and AE, since glutamate-mediated excitotoxicity takes place in many types of acute and chronic CNS diseases (126). Activation of microglia and presence of specific inflammatory proteins such as IL-6 and interleukin-8 (IL-8) in the CSF of psychiatric patients with schizophrenia or affective disorders support the role of CNS inflammation in these disorders (116).

### BSD and epilepsy

#### Clinical overlap

Bipolar spectrum disorders has been frequently described as rare in patients with epilepsy, due to anecdotical evidence rather than a standardized evaluation of the actual psychopathology (128). Nonetheless, growing evidence indicate an increasing prevalence of BSD and bipolar symptoms in people with epilepsy compared with that in the general population (128, 129). Moreover, psychogenic non-epileptic seizures (PNES), a subgroup of conversion disorders (CD) characterized by paroxysmal motor, non-motor, or behavioral alterations that resemble epileptic seizures without EEG correlates, are a common comorbidity of epilepsy and may be present in several psychiatric conditions, including BSD (130). A US survey reports manic symptoms to be present in 12.2% of patients with epilepsy, which is twice as common as in people with asthma and 7 times as common as in the healthy comparison group (128, 129, 131). A European study describes similar percentages (128, 132, 133). However, potential confounding variables should be considered. In most cases, bipolar symptoms occur around the ictal episode. Behavioral manifestations may precede (preictal) or follow (postictal) the ictal event, or they may represent the expression of the seizure activity (ictal) (128, 134, 135). An interictal dysphoric disorder (IDD) that may represent a phenotype copy of BSD has also been described (128, 132, 136). Manic symptoms may also be rare side effects of antiepileptic drugs (AEDs) (137, 138). AED-related manic symptoms may be due to the toxic effects of the drug or the forced normalization phenomenon (129). Currently, the link between the two conditions is still tenuous. However, considering the substantial burden of psychiatric disorders in epileptic patients, bipolar symptoms should be appropriately recognized and treated (128).

#### Converging biological pathways

Bipolar spectrum disorders and epilepsy have a similar clinical course, characterized by acute and paroxysmal episodes (128, 129, 139) treated with AEDs like carbamazepine, valproate, and lamotrigine (128, 137). It has been hypothesized that the kindling model represents the underlying pathophysiology of seizures and BSD. Moreover, the efficacy of several AEDs in BSD could be explained by similar modifications in neurotransmitters or the functioning of voltage-opened ion channels (129). Different studies suggest altered GABAergic-related mechanisms in BSD and the involvement of glutamate in stress-related neurotoxicity. The same neurochemical imbalance has been described as a relevant pathogenetic driver of epilepsy, possibly playing a similar role in both conditions (140). In patients with acute mania and BSD, an excess of the inward sodium current known to correlate with neuronal hyperexcitability and seizure facilitation has been described (128).

## Pathophysiology of BSD

### Genetic overlap

Bipolar spectrum disorders show a high degree of heritability (60–80% in twins studies) (141), while no mendelian inheritance pattern has been found. GWAS studies have identified more than 40 SNPs associated with an increased risk of development of BSD (16, 142). A recent GWAS analysis has found 87 enriched pathways that are relevant for BSD. Most are involved in cellular processes, signal transduction, metabolic processes, neuronal activities, immune system, and inflammation-related processes (143). Six of these metabolic pathways (drug metabolism, retinol metabolism, pentose and glucuronate interconversions, porphyrin and chlorophyll metabolism, starch and sucrose metabolism, ascorbate, and aldarate metabolism) were connected to dementia-related mechanisms, thereby suggesting links between BSD and the risk of dementia (143).

### Ion homeostasis

#### TRP channels

Calcium dysregulation has long been implicated in BSD pathophysiology, since intracellular calcium signaling is increased in BSD (144). Transient receptor potential (TRP) is a family of non-selective cation permeable channels, most of which show a low selectivity for Ca^2+^ ions. The receptor has been implicated in a wide array of CNS disorders, such as AD, PD, stroke, epilepsy, depression, bipolar disorder, and migraine, all associated with disturbances in Ca^2+^ homeostasis (145, 146). In BSD, TRP channels play an important role in disease development and progression. Genetic studies on BSD have shown that a mutation of TRPM2 gene reduces the dephosphorylation of GSK-3β, a crucial pathway involved in Ca^2+^ homeostasis (146, 147). Thus, TRPM2, as well as other TRP channels, may play a role in neurodegenerative disorders by interfering with Ca^2+^ homeostasis and mitochondrial activity, therefore activating the apoptosis pathways (146). TRP family is also involved in the etiology of epilepsy and in the generation of seizures (146). A knockout (KO) TRPM2 murine model showed reduced levels of neuroinflammation and neurodegeneration and increased epilepsy-induced psychological disorders, suggesting that TRPM2 favors the development and evolution of epilepsy-related brain injury (148).

#### Na^+^/K^+^-ATPase

Sodium–potassium ATPase (Na^+^/K^+^-ATPase) is an integral protein complex, responsible for ions transfer across the plasma membrane. In the healthy brain, almost 50% of the ATP consumption is due to the activity of this complex. Its structural or functional alterations impair neuronal excitability (149) and promote intracellular Ca^2+^ overloads (149, 150). It has been suggested that mood state-related reductions in Na^+^/K^+^-ATPase plays a central role in the pathophysiology of BSD (150).

In particular, allelic association between BD and a Na^+^, K^+^-ATPase α subunit gene (ATP1A3) has been reported (151). The decreased Na^+^/K^+^-ATPase activity in peripheral blood cells has been described during the acute phases of BSD (150, 151). Several studies have reported that intracerebroventricular injection of ouabain (a Na^+^/K^+^-ATPase inhibitor) induces hyperactive behavior in rats. Moreover, Li^+^ increases the Na^+^/K^+^-ATPase activity by reducing the formation of myoinositol and, as a consequence, attenuates the second messenger response (150). Several protein aggregates implicated in the pathophysiology of neurodegenerative disorders (amyloid-β, α-synuclein, and protein tau) directly interact with the Na^+^/K^+^-ATPase, in particular with its α3-subunit, and its activity may be reduced by this pathological conditions (149), leading to the development of manic disorders and, at the same time, to intracellular Ca^2+^ overload, that enhances the neurodegenerative process.

### Inflammation

During the last decades, growing evidence has supported the role of inflammation in the development of mood symptoms and BSD. The observation of the so-called “sickness behavior,” i.e., the behavioral changes observed in inflammatory conditions, characterized by changes in mood, social behavior, and cognition, has suggested a role of the immune system in the development of mood disorders (111, 152). Even the SARS-CoV-2 infection has been related with the development of manic episodes (153). Moreover, a certain degree of association between BSD and autoimmune diseases is known (107) and a prior hospitalization due to an autoimmune disease or infection has been recognized as a risk factor for developing a major mood disorder (111). Following this observational evidence, many studies have investigated the inflammatory state of patients with BSD, highlighting the presence of increased plasma levels of some pro-inflammatory cytokines (IL-1β, IL-6, and TNF-α) in BSD patients, compared to healthy controls (152), mainly during manic episodes. BSD also show higher levels of CRP compared to healthy controls in any stage of the disease. These levels increase more during the manic phase than during depression or euthymia (152, 154). Autoimmune pathologies (such as MS and systemic autoimmune diseases) and infections can trigger a condition of chronic low-grade inflammation. The mechanisms that can explain the neuropsychiatric alteration following infectious events are molecular mimicry, stress-induced microglial activation, disruption of the BBB, and alterations of epigenetic pathways (111). Chronic inflammation can lead to neurotoxicity and neurodegeneration as pro-inflammatory cytokines increase oxidative stress and promote microglial hyperactivation, thereby impairing neurotransmission and triggering neuronal loss (155). Post-mortem evaluations have revealed significant increases in excitotoxicity and neuroinflammatory markers in the prefrontal cortex of BSD patients, confirming the hyperactivation of the IL-1R receptor cascade and increases in NMDA receptor activity (155). The overlap between neuroinflammatory and psychiatric conditions is becoming so evident that some authors have conceptualized the notion of “autoimmune psychosis.” From this point of view, besides the clear autoimmune conditions defined in the spectrum of AE, it is possible to define a large number of psychiatric disorders as the expression of an overactivation of immune response that increases the risk of developing psychotic symptoms (111). Therefore, these authors have proposed a diagnostic algorithm to better identify immunologic alterations in patients with new-onset neuropsychiatric symptoms that involves screening for blood, CSF, and imaging markers (111).

### Neurotransmission: Dopaminergic and glutamatergic pathways

Excessive dopamine neurotransmission is involved in the development of manic and psychotic symptoms (156). Reduced dopamine transporter functioning, i.e., the impaired regulation of dopamine levels at synapses, recapitulates many aspects of BSD-related manic manifestations in preclinical models (157). Reduced plasma levels of dopamine-β-hydroxylase (DβH), an enzyme converting dopamine to norepinephrine, relate to the severity of BSD symptoms (158), and, compared to controls, striatal dopamine synthesis is increased in BSD patients with psychosis (159). The glutamatergic system has also been implicated in the pathophysiology of BSD (160, 161). The hyperactivation of these pathways can synergistically promote neuronal and non-neuronal stress, which leads to functional impairment and cell depletion. Dopamine has a high redox potential, reacts with nitrogen species, and increases oxidative stress (162). GABAergic transmission is decreased in BSD (140), thereby promoting glutamate/GABA imbalance with increased excitotoxicity (163) (164). Serotonin (5-HT) has been extensively associated with several psychiatric disorders, including BSD and ICD (165–167). Selective serotonin reuptake inhibitors (SSRIs) are the most used drugs for depressive mood disorders (168). Some evidence indicate a close interaction between the serotoninergic system and neurotrophic factors. For instance, BDNF promotes the development and optimal activity of serotoninergic neurons. At the same time, 5-HT controls and enhances BDNF expression (169), providing another functional crosslink between mood disorders and neurodegeneration.

## Conclusion

**FIGURE 2:**
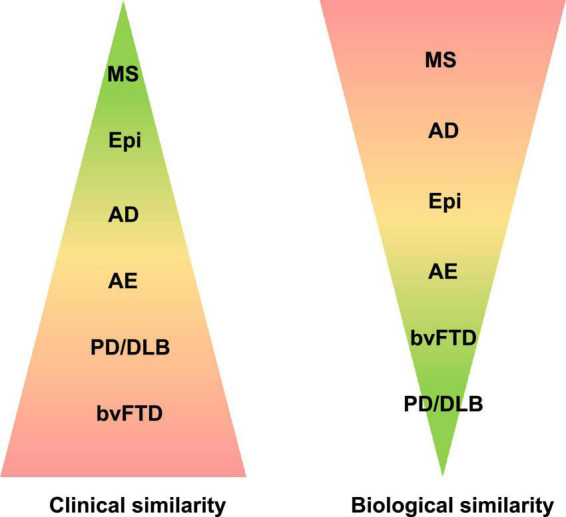
Neurological disorders of the central nervous system are here classified according to clinical and biological similarity to bipolar spectrum disorders (BSDs). Color coding and the corresponding area indicate the size of similarity. AD, Alzheimer’s disease; AE, autoimmune encephalitis; bvFTD, behavioral variant of frontotemporal dementia; Epi, epilepsy; MS, multiple sclerosis; PD/DLB, Parkinson’s disease, dementia with Lewy bodies.

**FIGURE 3:**
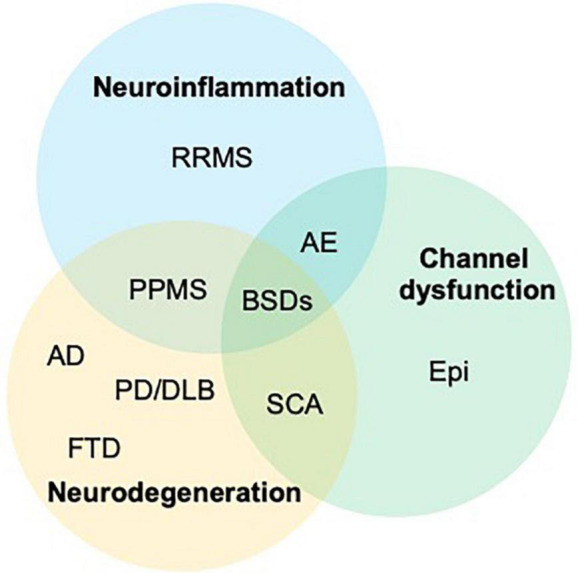
The graph outlines the intersections of three main pathological changes affecting the central nervous system and shows how all three are involved in the onset of bipolar spectrum disorders (BSDs). AD, Alzheimer’s disease; AE, autoimmune encephalitis; bvFTD, behavioral variant of frontotemporal dementia; Epi, epilepsy; PD/DLB, Parkinson’s disease, dementia with Lewy bodies; PPMS, primary progressive MS; RRMS, relapsing remitting multiple sclerosis; SCA, spinocerebellar ataxias.

Our review outlines the outstanding co-occurrence of Neurologic Disorders and BSDs (Figures 1–3).

Among the different studies, attention was addressed to possible common mechanism related to synaptic malfunctions (e.g., epilepsy), network disruption (e.g., MS), neurotransmitters imbalance (PD and dementias). Some studies attempted an investigation of possible phenotypes of Dementia associated with BSD.

For FTD and AE the occurrence of BSD symptoms was interpreted as a misdiagnosis of BSD, and this interpretation may be deemed acceptable, due to the prominent occurrence of psychosis in FTD (16, 27, 28) and to the short disease course in Encephalitis.

As this interpretation appears untenable for other Neurologic Disorders, the co-occurrence of BSD and Neurologic Disorders was, mostly, explained as a dependent on epidemiological overlaps, due to the prevalence of the disorders in general populations.

Bipolar spectrum disorders has a prevalence of around 5%, when considering plainly the three variants categorized by DSM-5 (3). PD has a prevalence of 2% in populations aged above 65, Dementias, also, have age-dependent prevalences, ranging from 5 to 20% or more according to age. The population prevalence of Epilepsy in its severe/refractory forms and of MS is around 0.05%. Therefore, in a gross estimate, 5% of patients with neurologic disorders would be expected to present with a history of BSD. Given the high prevalence of BSD, PD, and Dementia the epidemiological overlaps would result in a considerable number of patients sharing common features.

Therefore, in Neurologic Disorders, the co-occurrence was interpreted as a statistical effect (27, 70, 91), or random finding, until recent powerful epidemiology and meta-analysis studies outlined the coexistence of disorders, and the predictive value for the development of symptoms and response to treatments (16, 98, 129).

This statement is definitely valid for PD and Dementia (5, 20, 27, 87, 170–172), needs validation for Epilepsy and MS.

The evidence of an undeniable statistical correlation, suggests the necessity for different considerations, addressing practical and theoretical issues.

### Practical issues

The robust evidence of a phenotype including BSD symptoms for Neurologic Diseases, with emergence of manic conditions, and its corollaries, including ICDs, somatic symptoms disorders and addiction behaviors, suggests that some assumptions related to drug effects should be reconsidered with caution.

An outstanding example is provided by the history of dopamine agonist (DAs) treatments in PD.

Dopamine agonist induce psychosis and ICDs in a variable percentage of PD patients, ranging from 8 to 30%. ICD, as described in these patients, consisted of gambling, hypersexuality, paraphilias, shopping sprees, aggressive driving, and hoarding, addictive consumption of drugs, compulsory activities often termed “punding,” with a denomination borrowed from definitions of effects of cocaine addiction (173–177).

This effect of Das was described since the early introduction of ergolinic Das, like bromocriptine, cabergoline, or pergolide (174, 178–181), and was also described in patients receiving only L-dopa based treatments (182–185). It must be remembered also, that J Updike, in his “Rabbit Tetralogy” (186) described, masterly, the mood shift induced by L-dopa, with hypersexuality, at a time when L-dopa was still a new, “experimental” treatment. It was, however, with the introduction of non-ergolinic Das (pramipexole, ropinirole, and rotigotine, which, at difference with ergolinic Das, were not causing organ fibrosis and cardiac vegetations) that ICD induction reached a worldwide mediatic coverage (58, 187–191), with presentation of class actions, successfully claiming compensations from pharmaceutical companies and prescribers (medical doctors).

These actions ended definitively the research on Das, including apomorphine, a DA with wider receptor interactions than ergolinic and non-ergolinic Das (192).

Despite the grossly evident similarity of these Das effects with the behavioral (manic) shifts induced by antidepressants in BSD patients, the pharmacological mechanisms were not investigated, therefore there is no understanding of the reason why drugs scarcely, or not, acting on serotoninergic pathways should induce similar, or same, effects as drugs acting on serotonine reuptake.

This blurring by mediatic clamors, left many other questions unanswered, in example:

(1) On the precise pharmacological effects of Das, i.e., the question is whether the known presynaptic effect of Das (193–195), as Das do not act only at a post-synaptic level but also on the release of catecholamine quantums from presynaptic vesicles (193), is effected at a general presynaptic level on catecholamines, including serotonine, or it acts on the single neurotransmitter dopamine.
(2) On the effect, and dose requirements, of DAs in patients with severe depression, as a mood shift induction should be properly investigated, in order to provide new treatment options in other mental disorders (196–198).
(3) On the mechanism of DAs withdrawal syndrome (DAWS) (199), which consists of severe dysphoria with dysautonomia, observed in a minority of PD after the interruption of DAs treatments, and which is again similar to severe rebounds of depression in patients experiencing withdrawal of antidepressant drugs.

Moreover, there are no studies on the follow-up of patients who, putatively, experienced ICD because of DAs exposition, and it is not known, therefore, whether the effect was unique or linked to more complex predispositions.

The example of PD shows the disruptive effect of mediatic clamor on scientific inquiry, with its burden of unanswered questions, but other unanswered questions emerge when considering, among the other conditions, the co-occurrence of BSD and Dementia or Epilepsy.

Properly designed studies should address questions on antidepressant drugs effects and mechanisms in the subphenotypes expressing the association of disorders, in order to attempt the tailored approach to treatments.

### Theoretical issues

The coexistence of different neurologic disorders and bipolar disorder suggests that the behavioral patterns which are deemed proper of BSD are the result of predispositions to unstable control system acting at the network or at the synaptic level, giving origin to the cascade of symptoms as a final malfunctioning pathway. Similar malfunctions of the DMN are observed in psychotic episodes of FTD, PD, Dementias, some Epilepsies, and manic episodes with psychosis of BSD patients (78, 80, 81). The core malfunction results in disinhibition of PCC, which is released from the control of Frontal Cortex, possibly resulting in the activation of short distance connections (“small world” networks, as opposed to “large world” connections including the Fronto-Parietal Control network) (200, 201) and the emergence of resilient hallucinations, delusions and subcortical behaviors, like the impulsive ones linked to frontal lobes alterations. The hypothesis could be pushed forward, to hypothesize that BSD, in its most severe features, could represent the final phenotype of dysfunctions taking origins from different causative factors, but converging to a common eventual pathway. In this context, the current debate on the psychiatric classification system highlights the importance of adopting the “medical model” of disease categorization, which assumes that a disease is characterized by three components: an etiological agent, a pathological process, and symptoms/signs (202). This approach is deemed essential to strengthen the “scientific credibility” of psychiatry. Nowadays, the accepted systematization of psychiatric disorders is mainly based on the clinical presentation [DSM-5, (3)] and systematically leaves out the etiologic question. On the other hand, a growing amount of evidence from the neurobiological correlates of psychiatric conditions is setting the foundations for a more biological, and thus “valid” and “reliable,” approach to the field (203). In the future, the identification of a definite pathophysiological background will strengthen the nosographic understanding of BSD as a continuum and mitigate the common problem, in psychiatry, of low diagnostic validity, as juxtaposed to good diagnostic reliability (204, 205). Thus, future neurobiological and neuroimaging studies should focus on discovering a “BSD fingerprint,” which could help disentangle complex and overlapping clinical pictures and guide the formulation of future diagnostic criteria. As discussed in a recent paper (206), the Frontal lobe, due to its evolutional characteristics, is the weak point of human brain, as the size increment of the structure was not paralleled by size increments of brainstem structures providing the neurotransmitters supply (5, 206). With this hypothesis, the need for complex recategorization of BSD, such as proposed by Akiskal and Pinto, who provided examples of several BSD prototypes (13), finds a robust support, and opens new approaches to research.

## Author contributions

AD, PA, MO, and AT: conceptualization. AD, PA, and MO: methodology. AD, PA, MR, MO, and AT: writing—original draft preparation. AD, PA, MR, SS, MO, and AT: writing—review and editing. SS, MO, and AT: supervision. All authors contributed to the article and approved the submitted version.
